# Optimization of Sintering Conditions to Enhance the Dielectric Performance of Gd^3+^ and Ho^3+^ Codoped BaTiO_3_ Ceramics

**DOI:** 10.3390/molecules27217464

**Published:** 2022-11-02

**Authors:** Jianghui Bai, Qiaoli Liu, Xia Li, Xin Wei, Liping Li

**Affiliations:** 1 State Key Lab of Inorganic Syntheses and Preparative Chemistry, College of Chemistry, Jilin University, Changchun 130012, China; 2 Key Laboratory for Special Functional Materials at Jilin Provincial Universities, Jilin Institute of Chemical Technology, Jilin 132022, China; 3 Innovation Center for Chemical Science, College of Chemistry, Chemical Engineering and Materials Science, Soochow University, Suzhou 215123, China

**Keywords:** energy storage, sintering conditions, dielectric ceramics, Gd^3+^, Ho^3+^, BaTiO_3_

## Abstract

BaTiO_3_ dielectric capacitors, one of the important energy storage devices, play critical roles in storing electricity from renewable energies of water, wind, solar, etc. The synthesis of BaTiO_3_ ceramics with weak temperature dependence and a high dielectric constant at room temperature (*ε*_RT_′) is an urgent problem to meet the miniaturization and large capacity of dielectric capacitors. Doping rare earth elements into BaTiO_3_ can solve this problem, but it is still challenging. In this work, we adopt a synergistic strategy of increasing *ε*_RT_′ and improving the temperature stability by codoping Gd^3+^ and Ho^3+^, respectively, to address this challenge. By carefully adjusting the synthesis conditions in the solid-state reaction, codoping 7% Gd^3+^ and 7% Ho^3+^ in BaTiO_3_ (BGTH7) ceramics were synthesized. The temperature-dependent dielectric constant reveals that the obtained optimal BGTH7 ceramic satisfies the X7U specification and displays a stable *ε*′ in the temperature range of −55~125 °C. The optimal BGTH7 ceramic after sintering at 1400 °C for 6 h exhibits a high dielectric constant of 5475 and low dielectric loss (tan *δ*) of 0.0176, hitherto exhibiting the best performance in X7U ceramics. The findings in this work are conducive to the miniaturization and stabilization of dielectric energy storage devices.

## 1. Introduction

The limited amount of fossil fuels raise much attention about the development of renewable resources [1,2]. The conversion of renewable energy sources into electrical energy and efficient power storage are essential to solve the energy crisis [3,4]. Commonly used electrical energy storage devices are lithium batteries, supercapacitors, and dielectric capacitors. The low release power of lithium batteries and the instability of supercapacitors restrict their application in energy storage [5]. Among the dielectric energy storage materials, lead-free BaTiO_3_ dielectric capacitors are attracting attention for overcoming the above-mentioned disadvantages, exhibiting ultra-fast charge/discharge capability and high temperature tolerance [6,7].

The capacitance (*C*) of dielectric capacitors is governed by the dielectric constant (*ε*′), capacitance plate area (*S*), and distance between two pole plates (*d*), as shown in the formulas below:

(1) $$C=\varepsilon^{\prime }S/4\pi kd$$

k is the electrostatic force constant (k = 8.987551 × 10^9^ N·m^2^/C^2^). BaTiO_3_ dielectric ceramics are a key component of dielectric energy storage capacitors. However, the unstable and low capacity of BaTiO_3_ dielectric capacitors cannot meet the demand for miniaturization and the high performance of dielectric capacitors [8]. Therefore, obtaining a stable and high dielectric constant near the room temperature (*ε*′_RT_) for BaTiO_3_ ceramics can meet the requirements of miniaturization and high performance of dielectric capacitors, which are more widely used in miniaturized and integrated circuit systems [9,10].

In previous studies, significant efforts have been made to address the issues mentioned above. Tetragonal BaTiO_3_ has large spontaneous polarization and high *ε*′, eventually increasing the energy density [11,12]. Ma et al. prepared BaTiO_3_-La_2_O_3_-SiO_2_ particles. More La^3+^ and Si^4+^ ions incorporated into the BaTiO_3_ lattice with increasing SiO_2_ enhanced spontaneous polarization and resulted in increased energy density [13].

During the tetragonal to the cubic transition of BaTiO_3_ at 120 °C, *ε*′ increases sharply, forming a dielectric peak [14]. Shifting the dielectric peak temperature (*T*_m_) to room temperature contributes to high *ε*′_RT_. Gd-doped BaTiO_3_ can significantly reduce the *T*_m_ and increase *ε*_RT_′ [15,16]. Gd-doped 0.7BaTiO_3_-0.3Sr_0.2_Bi_0.7_TiO_3_ ceramics possess high *ε*′_RT_ (*ε*′_RT_ ≈ 4000) and *T*_m_ near −19 °C, exhibiting excellent energy storage. The difference in the valence of ions at the A-site induces a decreased tolerance factor, resulting in a decrease in *T*_m_ [17].

Moreover, the incorporation of Ho in BaTiO_3_ can broaden the dielectric peak and obtain stable dielectric constant [18]. In recent research reports, Hu [19] successfully synthesized Bi_2_O_3_ and Ho_2_O_3_ codoped BaTiO_3_-based ceramics by a solid-phase method, with a stable *ε*′ between 2400 and 2600 and satisfactory temperature stability of capacitance with an X8R dielectric specification. La-, Pr-, Nd-, Sm-, and Ho- codoped BaTiO_3_ all had wide dielectric peaks, satisfying the dielectric specifications of X5T, X6T, X7T, and X8S, respectively [20].

**Figure 1:**
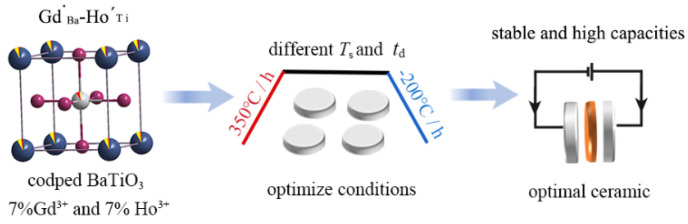
Target roadmap for high and stable capacitance achievement.

Although the dielectric performance was enhanced using the method reported above for BaTiO_3_, doping Gd or Ho alone is not sufficient to increase *ε*′_RT_ and stable *ε*′ in the temperature range of operation. The dielectric performance of doped ceramics is also dependent on sintering temperature (*T*_s_) and dwell time (*t*_d_). Therefore, we propose a synergistic strategy codoping 7% Gd^3+^ and 7% Ho^3+^ in BaTiO_3_ (BGTH7), carefully adjusting the synthesis conditions by the solid-phase method to obtain high *ε*′_RT_ and stable *ε*′, achieving stable and high capacities for BaTiO_3_ dielectric capacitors. The specific roadmap was shown in Figure 1.

In this work, the phase structure of BGTH7 ceramics was studied by X-ray diffraction and Raman spectroscopy. The improved performance with dielectric diffuse phase transition behavior can be obtained from dielectric measurement. The shift of *T*_m_ was caused by the charge-coupled substitution of Gd^3+^ and Ho^3+^ at both Ba and Ti sites, which was confirmed by X-ray photoelectron spectroscopy (XPS), electron paramagnetic resonance (EPR), and Raman spectrum. The scanning electric microscope (SEM) images show that optimizing *T*_s_ can improve the density and average grain size of the ceramic samples. Eventually, high *ε*′_RT_ and stable *ε*′ at work temperature can be obtained, which is promising for miniaturization and high performance of devices.

## 2. Results and Discussion

### 2.1. Effect of Sintering Temperature and Dwell Time on Phase Purity and Morphology

**Figure 2:**
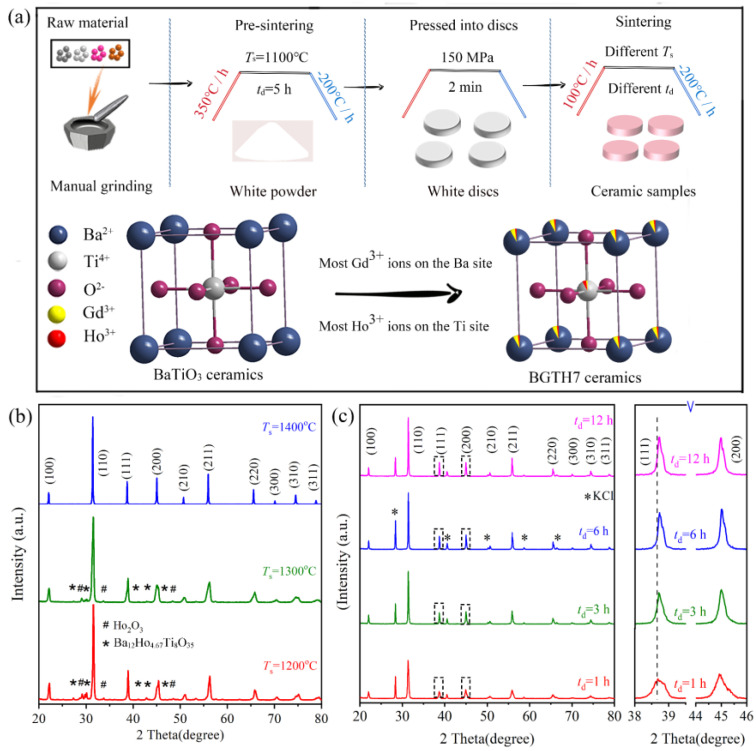
(**a**) Schematic illustration of the synthesis of BGTH7 ceramic samples. (**b**) X-ray diffraction patterns of the BGTH7 ceramics sintered at different sintering temperatures (*T*_s_) for 12 h. (**c**) X-ray diffraction patterns of BGTH7 ceramics sintered for different dwell times (*t*_d_) at 1400 °C. The right panel shows the enlarged diffraction peaks of (111) and (200) in the 2*θ* ranges of 38–39.5° and 44–46°, respectively.

Gd^3+^ and Ho^3+^ codoped BaTiO_3_ ceramics were synthesized by a solid-state method to achieve a high *ε*′_RT_ and stable *ε*′ in the operating temperature range. Usually, the sintering temperature (*T*_s_) and dwell time (*t*_d_) greatly affect the phase composition in solid-phase reactions. Thus, we systematically adjusted *T*_s_ and *t*_d_ to optimize the sintering conditions (Figure 2a). Two BGTH7 ceramics after sintering at *T*_s_ = 1200 and 1300 °C contained impurity phases Ba_12_Ho_4.67_Ti_8_O_35_ (PDF # 43−0420) and Ho_2_O_3_ (PDF # 43−1018) (marked with * and # in Figure 2b, respectively). Sintering at low temperatures makes it difficult to incorporate Ho^3+^ into the perovskite structure owing to the large radius of Ho^3+^ (0.901 Å) in octahedral coordination compared to that of Ti^4+^ (0.605 Å) [21]. And as shown in Figure S1, ceramics sintered at 1200 and 1300 °C exhibited low density and small grain size. High *T*_s_ can change the boundary of the phase diagram in multiple composition compounds but also increase the solution limit of doped ions in perovskite [22]. Thus, at *T*_s_ = 1400 °C, BGTH7 ceramics exhibited a pure phase of perovskite.

The phase structure of BGTH7 ceramics sintered at 1400 °C with different *t*_d_ was examined by XRD. BGTH7 ceramics sintered for different *t*_d_ (1, 3, 6, 12 h) are pure phases, as shown in Figure 2c. As *t*_d_ increased, the XRD peaks gradually narrowed, and their intensities were enhanced, demonstrating an improvement in crystallinity. To accurately determine the shift of the peak, the internal standard KCl was added to the BGTH7 ceramic powder. The enlarged peak at approximately 38° slightly shifted toward a higher 2*θ* angle as *t*_d_ increased, confirming a slight contraction in unit cell volume (*V*_0_). Notably, the diffraction peak (200) at approximately 45° for all ceramics did not show any splitting, which may be attributed to the cubic structure, similar to cubic BaTiO_3_ (PDF # 31−0174). Certainly, a slight distortion in the tetragonal phase structure or broadening of the XRD peaks can also obscure such a splitting, and this obscuring effect is difficult to detect by XRD. Therefore, in addition to XRD data, spectroscopic evidence could be helpful to prove the accurate phase structure of BGTH7 ceramics sintered at 1400 °C.

Raman spectroscopy can effectively distinguish the tetragonal or cubic phase of perovskite. Ideal cubic perovskite with *Pm*$\overset{—}{3}$*m* does not give any Raman signal due to a centrosymmetric structure belonging to the O_1h_ space group [23]. Once the BaTiO_3_ structure deviates from cubic symmetry, such as tetragonal with *P*4*mm*, four degenerate 3F_1*u*_ (IR) + F_2*u*_ (inactive) modes for the O_1h_ space group split into eight Raman active 3A_1_ + B_1_ + 4E modes [24]. Thus, the Raman signal can be observed.

**Figure 3:**
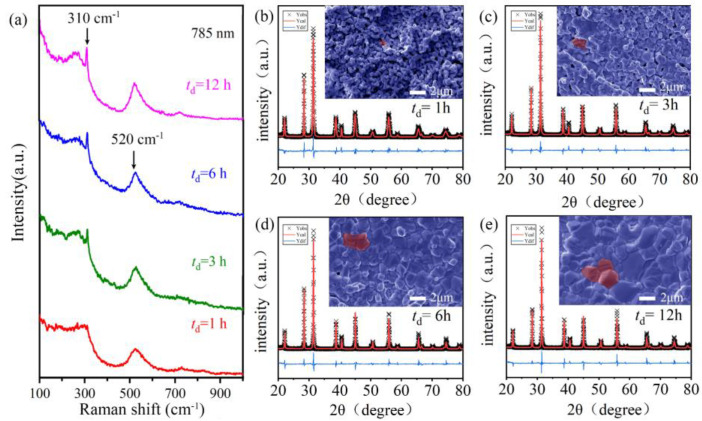
(**a**) Room-temperature Raman spectra in the low-wavenumber region of 100−1000 cm^−1^ for BGTH7 powder sintered for different *t*_d_ at *T*_s_ = 1400 °C. The results of the XRD refinement and SEM images for BGTH7 ceramics sintered with different *t*_d_ at *T*_s_ = 1400 °C. (**b**) *t*_d_ = 1 h, (**c**) *t*_d_ = 3 h, (**d**) *t*_d_ = 6 h, (**e**) *t*_d_ = 12 h.

As shown in Figure 3a, one peak at approximately 310 cm^−1^, usually from the B_1_ and E modes, was seen in the Raman spectra of sintered BGTH7 ceramics (*t*_d_ = 1 3, 6, 12 h), indicating that BGTH7 ceramics sintered at 1400 °C had a tetragonal structure [25,26]. Different from the sharp peak for the ceramics that underwent long dwell times, the peak at 310 cm^−1^ was not obvious for the BGTH7 ceramic sintered at *t*_d_ = 1 h due to incomplete grain growth. With these observations in mind, we refined the XRD data of BGTH7 ceramics sintered at 1400 °C by the tetragonal phase structure. The obtained lattice parameters in Table S1 show that the values of *a* and *c* are very close (*a* = *b* ≠ *c*, *α* = *β* = *γ* = 90°), i.e., the structure of BGTH7 ceramics sintered at 1400 °C slightly deviates from cubic symmetry. On the other hand, the Raman peak at approximately 520 cm^−1^ in Figure 3a corresponds to distorted (TiO_6_)^4−^ octahedra, confirming the presence of a small structure distortion. A similar distortion was also observed in previous reports [27,28]. The tetragonal phase structure of BGTH7 ceramics sintered at 1400 °C can have large spontaneous polarization and a high *ε′*, which is desirable for BaTiO_3_ dielectric energy storage capacitors [29,30].

The dwell time greatly affected the morphology of intact disc BGTH7 ceramics sintered at 1400 °C. According to the grain size analysis in Figure S2, the average grain sizes of the BGTH7 ceramics were approximately 0.71, 1.15, 2.11, and 3.17 μm for *t*_d_ = 1, 3, 6, and 12 h, respectively. As illustrated by the SEM images in the inset of Figure 3b–e, the BGTH7 ceramic after dwelling for *t*_d_ = 1 h exhibited tiny grains and pores, showing incomplete grain growth. The grain size was improved by prolonging the dwell time. These gradually increased grains filled the pores, resulting in a denser microstructure. The relative density (*ρ*_r_) of the BGTH7 ceramics was 73, 79, 86, and 93%, sintered at 1400 °C for *t*_d_ = 1, 3, 6, and 12 h, respectively, in which *ρ*_r_ was estimated using the following formula:

(2) $$\rho_{r}{=\rho }_{a}/\rho_{0}$$

*ρ*_0_ is the theoretical density obtained from Rietveld refinement, and *ρ*_a_ is the actual bulk density that can be measured using the Archimedes method.

### 2.2. Effect of Dwell Time on the Site Occupation of BGTH7 Ceramics Sintered at 1400 °C

Rare earth ions, such as Gd^3+^ and Ho^3+^, have different ion radii than Ba^2+^ and Ti^4+^, which often leads to their preferential occupation once they are doped in BaTiO_3_. Such a preferential occupation has an important influence on the dielectric property. In addition to the ionic radius, some other factors can also change the occupation of rare earth ions in the perovskite, such as the valence state of Ti and oxygen vacancies, which can be determined by XPS, EPR, and photoluminescence analysis.

**Figure 4:**
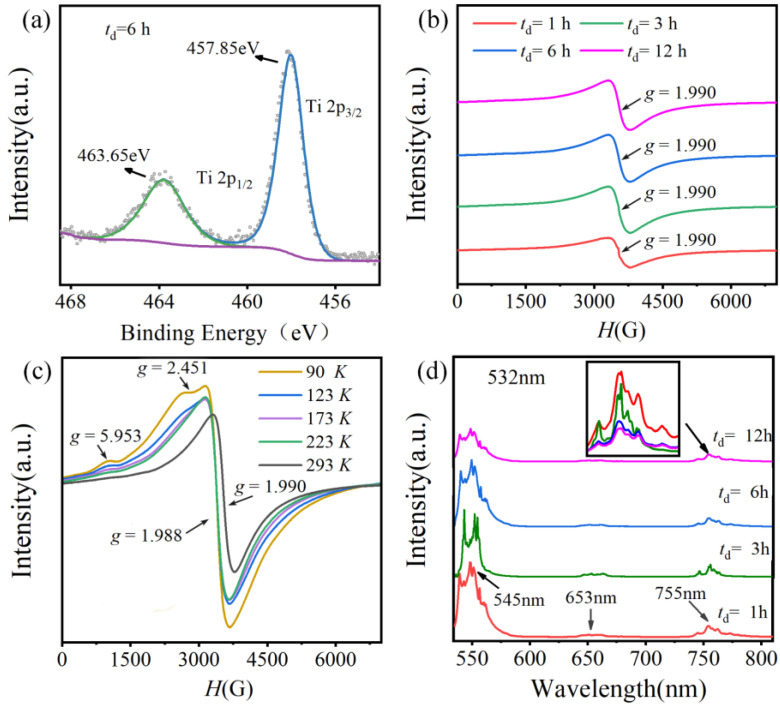
(**a**) Ti 2p XPS spectrum of BGTH7 sintered at *T*_s_ = 1400 °C for 6 h. (**b**) EPR spectra of BGTH7 sintered at *T*_s_ = 1400 °C measured at room temperature. (**c**) Temperature-dependent EPR spectra of BGTH7 sintered at *T*_s_ = 1400 °C for 6 h. (**d**) Raman spectra under 532 nm excitation of BGTH7 ceramics sintered at *T*_s_ = 1400 °C for 1, 3, 6 and 12 h.

The valence state of Ti ions was investigated by XPS. Figure 4a and Figure S3 shows the Ti 2p core level spectra of BGTH7 sintered at *T*_s_ = 1400 °C. Well spin-orbital splitting peaks Ti2p_3/2_ (at approximately 457.8 eV) and Ti2p_1/2_ (at approximately 463.6 eV) were observed, and the splitting energy of 2p_3/2_ and 2p_1/2_ was 5.8 eV, confirming the presence of Ti^4+^ ions [31,32]. Generally, the characteristic peak of Ti^3+^ 2p_3/2_ appears at approximately 455 eV [33]. Deconvolution analysis showed that there was the absence of a peak at 455 eV, proving the absence of Ti^3+^, i.e., only one oxidation state Ti^4+^ in the BGTH7 ceramics sintered at 1400 °C.

Due to the charge difference between dopant ions Gd^3+^/Ho^3+^ and host ions Ba^2+^ and Ti^4+^ in BaTiO_3_, the incorporation of Gd^3+^/Ho^3+^ could introduce a positive or negative charge center. These charge mismatches can be compensated by vacancies, described as follows by the Kröger–Vink notation:

(3) $${\mathrm{Ln}}_{2}O_{3}\overset{{\mathrm{BaTiO}}_{3}}{\to }2{\mathrm{Ln}}_{\mathrm{Ba}}^{•}+V_{\mathrm{Ba}}^{’’}+3O_{O}^{x}$$

(4) $${\mathrm{Ln}}_{2}O_{3}\overset{{\mathrm{BaTiO}}_{3}}{\to }2{\mathrm{Ln}}_{\mathrm{Ti}}^{\;’}+V_{O}^{••}+3/2O_{2}\left(g\right)$$

Here, Ln represents Gd^3+^ or Ho^3+^. $V_{\mathrm{Ba}}^{’’}{\;\mathrm{and}\;V}_{O}^{••}$ usually displays EPR signals at *g* = 1.974 and 1.955 [34,35]. In Figure 4b, only a broad and symmetrical signal with *g* = 1.990 was observed for BGTH7 ceramics sintered at 1400 °C. This signal is attributed to Gd^3+^ with an electron configuration of 4f^7^ (ground state is ^8^S_7/2_) [36]. The EPR spectrum of Gd^3+^ is highly dependent on the symmetry of ion coordination in Figure S4. New weak signals appeared with *g* = 5.953 and 2.451 at temperatures below 223 K in Figure 4c, which is associated with the change in Gd^3+^ symmetry from slightly distorted cubic coordination to intermediate coordination because of the increased structure distortion at a low temperature [37,38]. The absence of a signal related to $V_{\mathrm{Ba}}^{’’}{\;\mathrm{or}\;V}_{O}^{••}$ indicates the absence of vacancies. As is well known, performing EPR operations under ultrahigh vacuum and at low temperatures can increase the detection limit. Variable temperature EPR spectroscopy was investigated for the BGTH7 ceramic sintered at 1400 °C for 6 h. As illustrated in Figure 4c, the vacancy signals were not activated when the temperature was changed through *T*_m_ (270.36 K). Therefore, the incorporation of Gd^3+^ and Ho^3+^ could adopt a charge-coupled substitution on both the Ba and Ti sites:

(5) $${\mathrm{Ln}}_{2}O_{3}\overset{{\mathrm{BaTiO}}_{3}}{\to }{\mathrm{Ln}}_{\mathrm{Ba}}^{•}+{\mathrm{Ln}}_{\mathrm{Ti}}^{’}+3O_{O}^{x}$$

Ho^3+^ ions in some perovskites exhibit a strong photoluminescence emission. The Raman spectrum of Ho^3+^ doped BaTiO_3_ under 532 nm excitation is often used to probe the site occupations of Ho^3+^ ions because the concentration of Ho^3+^ ions on the Ba sites is highly dependent on the transition intensities of ^5^F_4_/^5^S_2_→^5^I_8_ at 545 nm, ^5^F_5_→^5^I_8_ at 653 nm, and ^5^F_4_/^5^S_2_→^5^I_7_ at 755 nm [21,39]. Figure 4d and Figure S5 display the Raman spectra of BGTH7 ceramics sintered at 1400 °C under 532 nm excitation. Taking the transition intensity at 755 nm as a comparison for the inset in Figure 4d, the concentration of Ho^3+^ at the Ba site can be estimated [17]. For BGTH7 ceramics with *t*_d_ values of 1, 3, 6, and 12 h, the contents of Ho^3+^ substituted for Ba^2+^ were 0.0059, 0.0050, 0.0022, and 0.0018, respectively. Increasing the dwell time at a high temperature (1400 °C) could reduce the incorporation of Ho^3+^ into the Ba site. The cross-site occupancy between Gd^3+^ and Ho^3+^ is described in Equation (5) and occurred in BGTH7 ceramics sintered at 1400 °C. Therefore, for BGTH7 ceramics with *t*_d_ values of 1, 3, 6, and 12 h, the contents of Ho^3+^ substituted for Ti^4+^ were 0.0641, 0.065, 0.0678, and 0.0682, respectively. From the ionic radius in Table S2, Gd^3+^ ions preferentially incorporated into the Ba site, while Ho^3+^ preferentially incorporated into the Ti site, forming a ${\mathrm{Gd}}_{\mathrm{Ba}}^{•}$-${\mathrm{Ho}}_{\mathrm{Ti}}^{’}$ complex. On the other hand, a small quantity of Ho^3+^ and Gd^3+^ change their occupation on the Ba site and Ti site to ${\mathrm{Ho}}_{\mathrm{Ba}}^{•}$-${\mathrm{Gd}}_{\mathrm{Ti}}^{’}$ according to photoluminescence analysis. When the sintering conditions were *T*_s_ = 1400 °C and *t*_d_ = 6 h, the real molecular formula of the obtained ceramic was (Ba_0.93_Gd_0.0678_Ho_0.0022_)(Ti_0.93_Ho_0.0678_Gd_0.0022_) O_3_, having more ${\mathrm{Gd}}_{\mathrm{Ba}}^{•}$-${\mathrm{Ho}}_{\mathrm{Ti}}^{’}$ complexes.

### 2.3. Dielectric Properties of BGTH7 Ceramics Sintered at 1400 °C

The Electronic Industry Association (EIA) classifies class II ceramic capacitors into two categories: capacitance-stable and high-capacitance ceramic capacitors. The most commonly used capacitance-stable capacitors are R-type capacitors. Over the operating temperatures, the capacitance change of R-type capacitors does not exceed 15%. However, these capacitors have relatively low dielectric constant, which are not suitable for miniaturized applications in electronic products. Another capacitor is the Y-type capacitor. Even though the Y-type capacitor has a high capacitance compared to the R-type capacitor, it is difficult to apply because of its unstable dielectric constant. A U-type capacitor combines the advantages of temperature stability and high capacitance, eventually having a stable and high dielectric constant over the entire operating temperature range [40,41,42,43].

**Figure 5:**
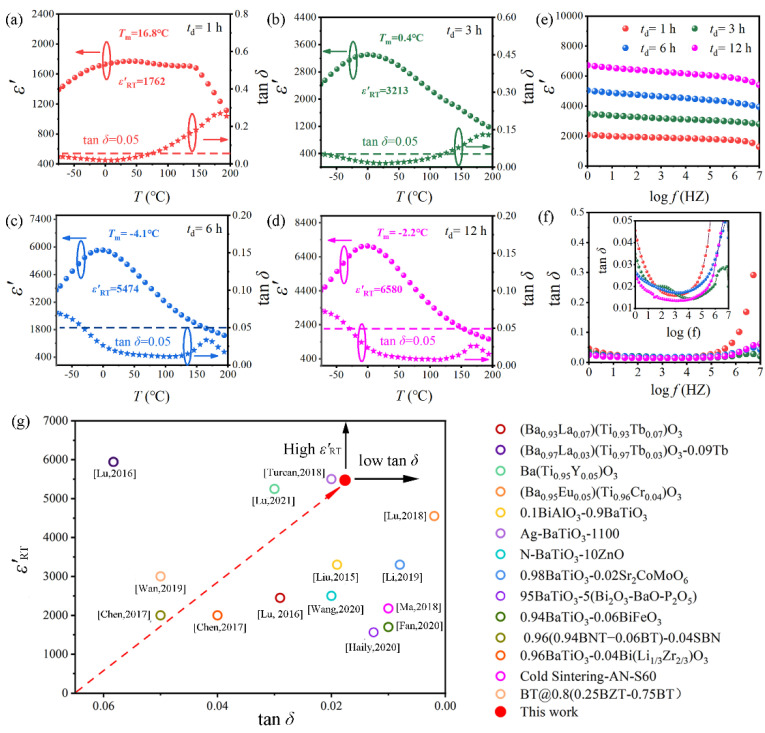
Temperature dependence of the dielectric constant (*ε′*) and dielectric loss (tan *δ*) measured at 1 kHz for BGTH7 ceramics sintered at *T*_s_ = 1400 °C for different *t*_d_. (**a**) *t*_d_ = 1 h, (**b**) *t*_d_ = 3 h, (**c**) *t*_d_ = 6 h, (**d**) *t*_d_ = 12 h. The frequency dependence of the dielectric constant (*ε′*) and dielectric loss (tan *δ*) was measured at room temperature for BGTH7 ceramics sintered at *T*_s_ = 1400 °C for different *t*_d_ (**e**) and (**f**). (**g**) The dielectric properties of this work are compared with those of previously reported BaTiO_3_-based dielectric ceramics with X7U specifications [2,44,45,46,47,48,49,50,51,52,53,54,55].

Table S3 lists EIA classification of Class II capacitors. Figure 5a–d shows the temperature dependence of the *ε′* and dielectric loss (tan *δ*) measured at 1 kHz for BGTH7 ceramics sintered at 1400 °C. According to Table S3, BGTH7 ceramics (*t*_d_ = 1, 3, 6, 12 h) satisfy the X8R, X7U, X7U, and X6U specifications, respectively. The dielectric properties for all BGTH7 ceramics are shown in Table S4. In particular, the BGTH7 ceramic sintered for 6 h at 1400 °C exhibits high *ε*′_RT_ (*ε*′_RT_ > 5000) and ultralow tan *δ* (tan *δ* < 0.02), with the location of the dielectric peak near the room temperature. This finding is very important for solving the problem of the location of the dielectric peak for conventional BaTiO_3_ ceramics being too high (close to 125 °C) to satisfy the requirement of the development and application of small electronics at room temperature.

*ε*′ and tan *δ* of BGTH7 ceramics sintered at 1400 °C with different *t*_d_ exhibited the same changes with frequency (Figure 5e,f). The value of *ε*′ in the high frequency region is lower than the low frequency region. The variation in *ε*′ with frequency can be interpreted by the Maxwell–Wagner’s two-layer models. The total polarization contributes to a high *ε*′ value at low frequency, while in the high-frequency region, dipole inversion fails to keep up with the changing electric field, causing *ε*′ to decrease.

Ceramics sintered at 1200 and 1300 °C exhibited low dielectric constant and high tan *δ*, as shown in Figures S6 and S7. Dielectric measurements demonstrated that the optimal BGTH7 ceramic sintered at T_s_ = 1400 °C and *t*_d_ = 6 h possesses high *ε*′_RT_ (*ε*′_RT_ = 5475) and low tan *δ* (tan *δ* = 0.0176) at 1 kHz, as well as enhanced frequency stability from 1 to 10^7^ Hz. In addition, this BGTH7 ceramic satisfied the X7U specification according to Table S4, having stable *ε*′ in the operating temperature range (−55~125 °C). As shown Table S5 and Figure 5g, the BGTH7 ceramic is superior to the X7U specification reported previously, having potential application in microminiature and temperature-stable BaTiO_3_ dielectric energy storage capacitors.

### 2.4. Understanding the Excellent Dielectric Properties of BGTH7 Ceramics Sintered at 1400 °C

#### 2.4.1. High Dielectric Constant at Room Temperature

*ε*′_RT_ gradually increases from 1762 to 6580 as the average grain size changes from 0.71 to 3.17 μm (*ρ*_r_ from 73% to 93%, respectively), as shown in Figure S8. The reason for increasing *ε*′_RT_ can be understood from the following two points: (1) Air acts as a stress snubber and reduces the entrapment forces between grains in the BaTiO_3_ ceramic, resulting in the formation of pores. Polarization is a critical factor in improving the dielectric constant. Normally, polarization is weakened by the depolarization field that occurs near the pores, resulting in decreasing dielectric constant [56]. High *ρ*_r_ shields the depolarization field, resulting in a high *ε*′ of ceramics. Among the four examined BGTH7 ceramics sintered at 1400 °C, the one sintered at *t*_d_ = 12 h, having *ρ*_r_ as high as 93%, gives the highest *ε*′_RT_ of 6580. (2) An easier and more regular motion of the domain wall can also increase *ε*′_RT_ [57,58]. Generally, large grains are conducive to forming orderly dipole alignment and promoting regular domain wall motion, thus increasing *ε*′. As the grain sizes increased, the dielectric peak was gradually enhanced, resulting in an increased dielectric constant (*ε*′_RT_) at room temperature. Therefore, a denser structure and large grain size are beneficial to obtaining high *ε*′_RT_ in BGTH7 ceramics sintered at 1400 °C.

#### 2.4.2. Shift of the Dielectric Peak

The Goldschmidt tolerance factor (*t*) can predict the symmetry of ABO_3_ perovskites that significantly affect their dielectric performance and is defined as the ratios of the constituent ionic radii of A, B, and O:

(6) $$t{=r}_{A}{+r}_{O}\;/\;\sqrt{2}{\;(r}_{B}{+r}_{O})$$

For pure BaTiO_3_, *r*_Ba_^2+^, *r*_Ti_^4+^, and *r*_O_^2−^ are 1.61 (in 12 coordinate), 0.605 (in octahedral coordination), and 1.4 Å; thus, *t* ≈ 1.06. A previous investigation has shown that the dielectric peak temperature (*T*_m_) of BaTiO_3_ is highly dependent on the tolerance factor (*t*). Reducing *t* can shift the *T*_m_ to a lower temperature [59]. When some Gd^3+^ ions are incorporated into Ba sites, the decrease in *t* shifts *T*_m_ to a low temperature. Instead, the substitution of Ho^3+^ for Ti^4+^ ions leads to *T*_m_ moving toward higher temperatures, similar to the incorporation of Ca^2+^ [21]. In other words, the charge-coupled substitution of Gd^3+^ and Ho^3+^ at both Ba and Ti sites (Equation (5)) has different effects on *T*_m_, which was caused by the different effects of substitution for Gd^3+^ and Ho^3+^ on Ti-O octahedral distortion [60]. The photoluminescence analysis in Figure 4d indicated that the Ho^3+^ content in the Ti site increases. Consequently, *T*_m_ first decreased and then increased, as shown in Figure S9.

All studied BGTH7 ceramics sintered at 1400 °C exhibit dielectric diffusion phase transition (DPT) behavior. The broadening dielectric peak was related to the grain size, which is consistent with numerous past reports [61,62]. In addition, Gd^3+^ and Ho^3+^ codoped BaTiO_3_ ceramics can also broaden the dielectric peak, similar to La-, Pr-, Nd-, Sm-, and Ho- codoped BaTiO_3_ ceramics [20]. Codoping of Ho^3+^ with Gd^3+^ has an important effect on the stability of *ε*′ over a wide temperature range.

**Figure 6:**
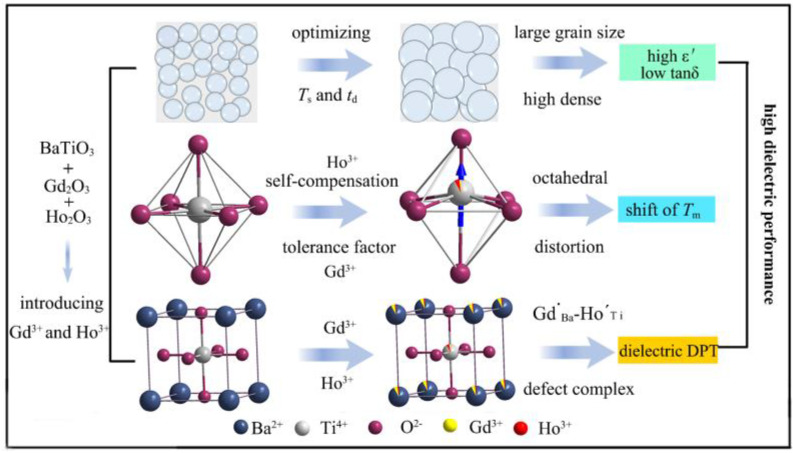
The mechanisms for improving dielectric properties.

The detailed mechanisms for improving dielectric properties is illustrated in Figure 6. Charge-coupled substitution of Gd^3+^ and Ho^3+^ at both Ba and Ti sites, decrease of tolerance factor, distortion of octahedral, as well as increased density can be optimized by adjusting sintering temperature, and time. Eventually, the optimal BGTH7 ceramic not only exhibited high *ε*′_RT_ but also dielectric DPT, which can ensure stable operation for BaTiO_3_ dielectric energy storage capacitors.

## 3. Materials and Methods

BaCO_3_ (99.5%), TiO_2_ (99.5%), Ho_2_O_3_ (99.95%), and Gd_2_O_3_ (99.90%) were used as raw materials, weighed according to 7% Gd^3+^ and 7% Ho^3+^ codoping in BaTiO_3_ (BGTH7), and milled. After drying and calcining at 1100 °C for 5 h for decarburization, the obtained powder was reground and pressed into discs with a diameter of 12 mm and a thickness of 2 mm at 150 MPa using an aqueous solution of PVA (12% by mass) as a binder. The final sintering conditions to form the BGTH7 ceramics were chosen as heating up directly from room temperature to different *T*_s_ (1200 °C ≤ *T*_s_ ≤ 1400 °C, *t*_d_ = 12 h) and different *t*_d_ (1 h ≤ *t*_d_ ≤ 12 h, *T*_s_ = 1400 °C) in the air at a heating rate of 100 °C/h, a cooling rate of −200 °C/h to 700 °C, and then furnace cooling to room temperature.

The crystallographic structures were studied by using X-ray diffraction (XRD: DX-2700, Dandong Haoyuan, Dandong, China) with Cu Kα_1_ radiation (*λ* = 1.540562 Å). The microstructure of the sintered samples was observed using an EVOMA 10 scanning electric microscope (SEM: EVO MA10, Zeiss, Oberkochen, Germany) operated at 15 kV. The conducting Au atoms were sputtered on the specimen surface for SEM observations. XRD Rietveld refinements were conducted with the General Structure Analysis System (GSAS) program. The 532 nm and 785 nm lasers were used for excitation to obtain the Raman spectra (RS) of the ceramics and photoluminescence (PL) of Ho^3+^ using a LabRAM XploRA Raman spectrometer (Horiba Jobin Yvon, Longjumeau, France). X-ray photoelectron spectroscopy (XPS, ESCA-LAB250) measurements were performed to study the valence states of cations. The measurement curves of XPS data were fitted by a mixed Gaussian–Lorentzian function, and Shirley-type background subtraction was used. EPR measurements were carried out with an X-band (≈9.4 GHz) spectrometer (A300, Bruker, Rheinstetten, Germany) at 90–298 K. The gyromagnetic constant (*g*) was calculated using *hv* = *gμ*_0_*H*, where *h* is the Planck constant (*h* = 6.626 × 10^−34^ J·s), *v* is the microwave frequency, *μ*_0_ is the Bohr magnetron (*μ*_0_ = 9.262 × 10^−24^ J/T), and *H* is the magnetic field strength. The dielectric properties of the ceramic samples were measured on a broadband dielectric spectrometer (Concept 41, Novocontrol Technologies, Montabaur Germany) in a temperature range of 198–473 K and a frequency range of 1 Hz–10 MHz.

## 4. Conclusions

Converting renewable energy to electricity and efficient electricity storage are keys to addressing the energy crisis. BaTiO_3_ dielectric capacitors are critical energy storage devices due to their ultrafast charge/discharge ability, exceptional cycle life and high-temperature tolerance. However, the unstable and low dielectric constant (*ε*′_RT_ = 1600) near the room temperature of BaTiO_3_ ceramics cannot meet the needs of miniaturization and the high capacity of dielectric capacitors. In this work, Gd^3+^ and Ho^3+^ codoped tetragonal BaTiO_3_ ceramics were prepared by carefully adjusting the synthesis conditions to address this issue. The optimal ceramic sintered at 1400 °C for 6 h had an ultrahigh room-temperature dielectric constant (*ε*′_RT_ = 5475) with low dielectric loss (tan *δ* = 0.0176). In addition, the BGTH7 ceramic exhibits good X7U dielectric properties in the temperature range of −55~125 °C, which is superior to other X7U BaTiO_3_-based ceramics reported previously. The mechanisms for improving dielectric properties can be attributed to the tetragonal perovskite structure, large grain size, and the formation of the ${\mathrm{Gd}}_{\mathrm{Ba}}^{•}$-${\mathrm{Ho}}_{\mathrm{Ti}}^{’}$ complex. The high capacitance of this ceramic helps reduce the capacitor size, improve the efficiency, and enable miniature applications. The BGTH7 ceramic can be prepared as dielectric capacitors with high capacitance, is widely used in pulse power devices, hybrid automotive power supplies, and other fields, and will be beneficial to many portable electronic applications. In addition, the ceramic exhibits wide temperature range stability and is suitable for applications in extreme environments such as polar, high altitude, and underground tunnels.

## Data Availability

Not applicable.

## Supplementary Materials

The following supporting information can be downloaded at: https://www.mdpi.com/article/10.3390/molecules27217464/s1, Figure S1: SEM images of the surface for BGTH7 ceramics with different *T*_s_ (a) *T*_s_ = 1200 °C, (b) *T*_s_ = 1300 °C, (c) *T*_s_ = 1400 °C for 12 h.; Figure S2: The grain size analysis of the BGTH7 ceramics of BGTH7 ceramics sintered for (a) 1 h (b) 3 h and (c) 12 h at 1400 °C; Figure S3: Ti 2p XPS spectra of BGTH7 ceramics sintered for (a) 1 h (b) 3 h (c) 12 h at 1400 °C; Figure S4: EPR spectra of BGTH7 ceramics sintered at different *T*_s_ for 12 h measured at room temperature; Figure S5: Raman spectra under 532 nm excitation of ceramics sintered at different *T*_s_ for 12 h measured at room temperature; Figure S6: Temperature dependence of the dielectric constant (*ε′*) and the dielectric loss (tan *δ*) for ceramics sintered at *T*_s_ = 1200 °C, 1300 °C and 1400 °C; Figure S7: Frequency dependence of the dielectric permittivity (*ε′*) and the dielectric loss (tan *δ*) for BGTH7 ceramics sintered at *T*_s_ = 1200 °C, 1300 °C and 1400 °C; Figure S8: *ε’*_RT_ and tan *δ* at room temperature vary with grain size (GS) and relative density (*ρ*_r_) for BGTH7 ceramics sintered at 1400 °C; Figure S9: Dielectric peak temperature varies with dwell time for BGTH7 ceramics sintered at 1400 °C; Table S1: Rietveld Refined lattice parameters of BGTH7 ceramics sintered at 1400 °C; Table S2: Ionic radii as a function of coordinate number (CN); Table S3: EIA Definition of Class II Capacitors; Table S4: Dielectric properties of all BGTH7 ceramics; Table S5: The dielectric properties of this work are compared with those of previously reported BaTiO_3_-based dielectric ceramics with X7U specifications.
