# Supplementary material for: Optimization of Sintering Conditions to Enhance the Dielectric Performance of Gd3+ and Ho3+ Codoped BaTiO3 Ceramics
Source: Molecules. 2022 Nov 2;27(21):7464. doi: 10.3390/molecules27217464 (PMC9653661; doi:10.3390/molecules27217464)
Supplement: Supplementary file 1 [file molecules-27-07464-s001.zip › molecules-1960096-supplementary.pdf]

molecules

Supporting Information

Article

Optimization of Sintering Conditions to Enhance the Dielectric Performance of Gd^{3+} and Ho^{3+} Codoped $BaTiO_3$ Ceramics

Jianghui Bai [1], Qiaoli Liu [2], Xia Li [3], Xin Wei [2] and Liping Li [1,*]

[1] State Key Lab of Inorganic Syntheses and Preparative Chemistry, College of Chemistry, Jilin University, Changchun 130012, China
[2] Key Laboratory for Special Functional Materials at Jilin Provincial Universities, Jilin Institute of Chemical Technology, Jilin city 132022, China
[3] Innovation Center for Chemical Science, College of Chemistry, Chemical Engineering and Materials Science, Soochow University, Suzhou 215123, China
* Correspondence: author: lipingli@jlu.edu.cn

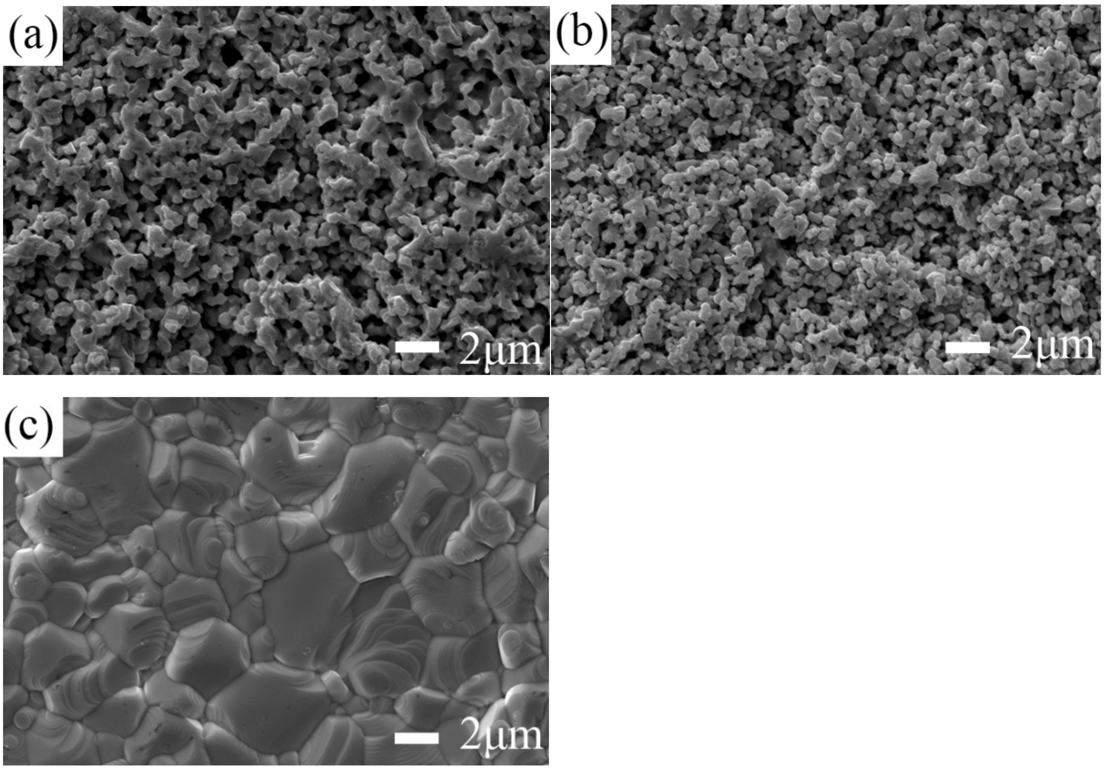

Figure S1. SEM images of the surface for BGTH7 ceramics with different T_s (a) T_s = 1200 °C, (b) T_s = 1300 °C, (c) T_s = 1400 °C for 12 h.

When T_s = 1200 °C and 1300 °C, there were many pores between the grains. This structure brought high dielectric loss (tan δ) and low dielectric constant (ε'_{RT}) at room temperature. Instead, a dense microstructure with increasing average grain size was observed for the BGTH7 ceramic sintered at 1400 °C for 12 h.

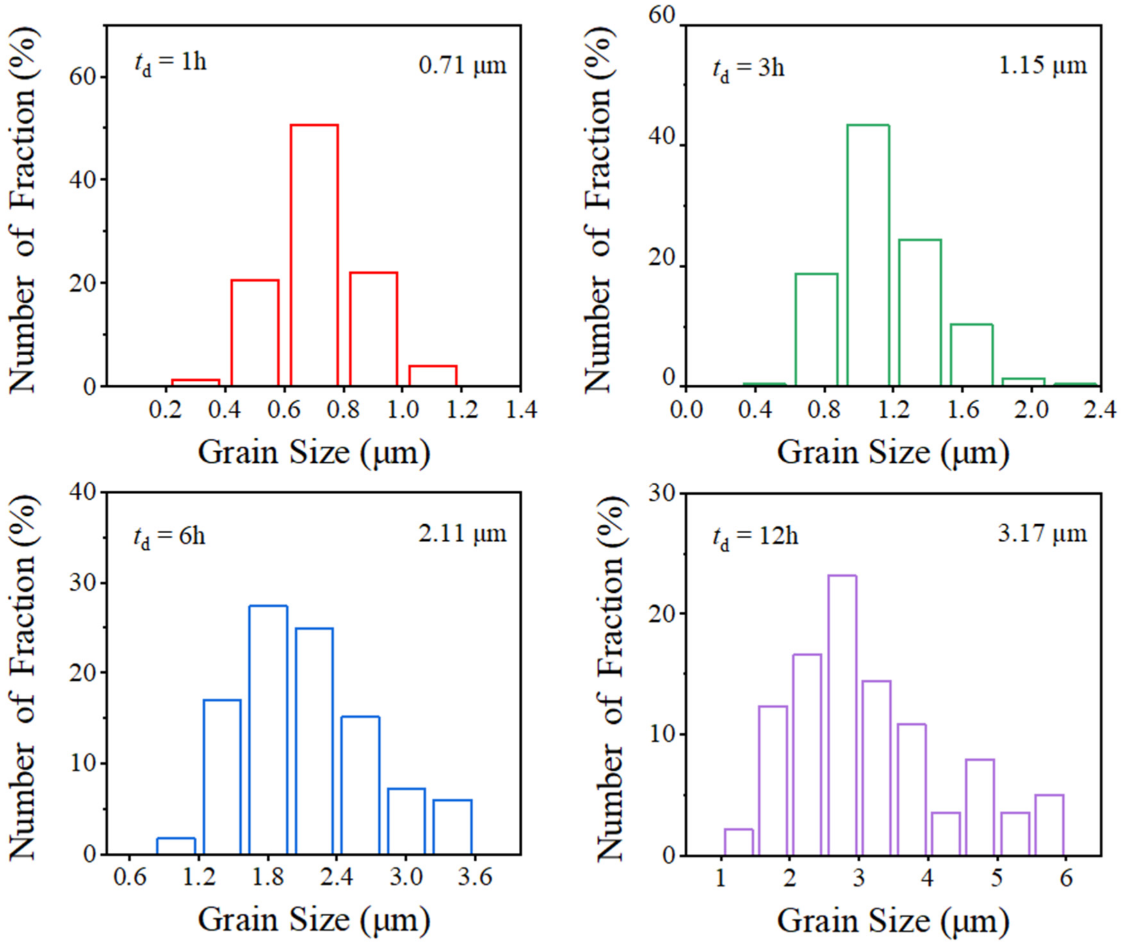

Figure S2. The grain size analysis of the BGTH7 ceramics of BGTH7 ceramics sintered for (a) 1 h (b) 3 h and (c) 12 h at 1400 °C

The average grain sizes of the BGTH7 ceramics were approximately 0.71, 1.15, 2.11 and 3.17 μm for t_d =1, 3, 6, and 12 h, respectively.

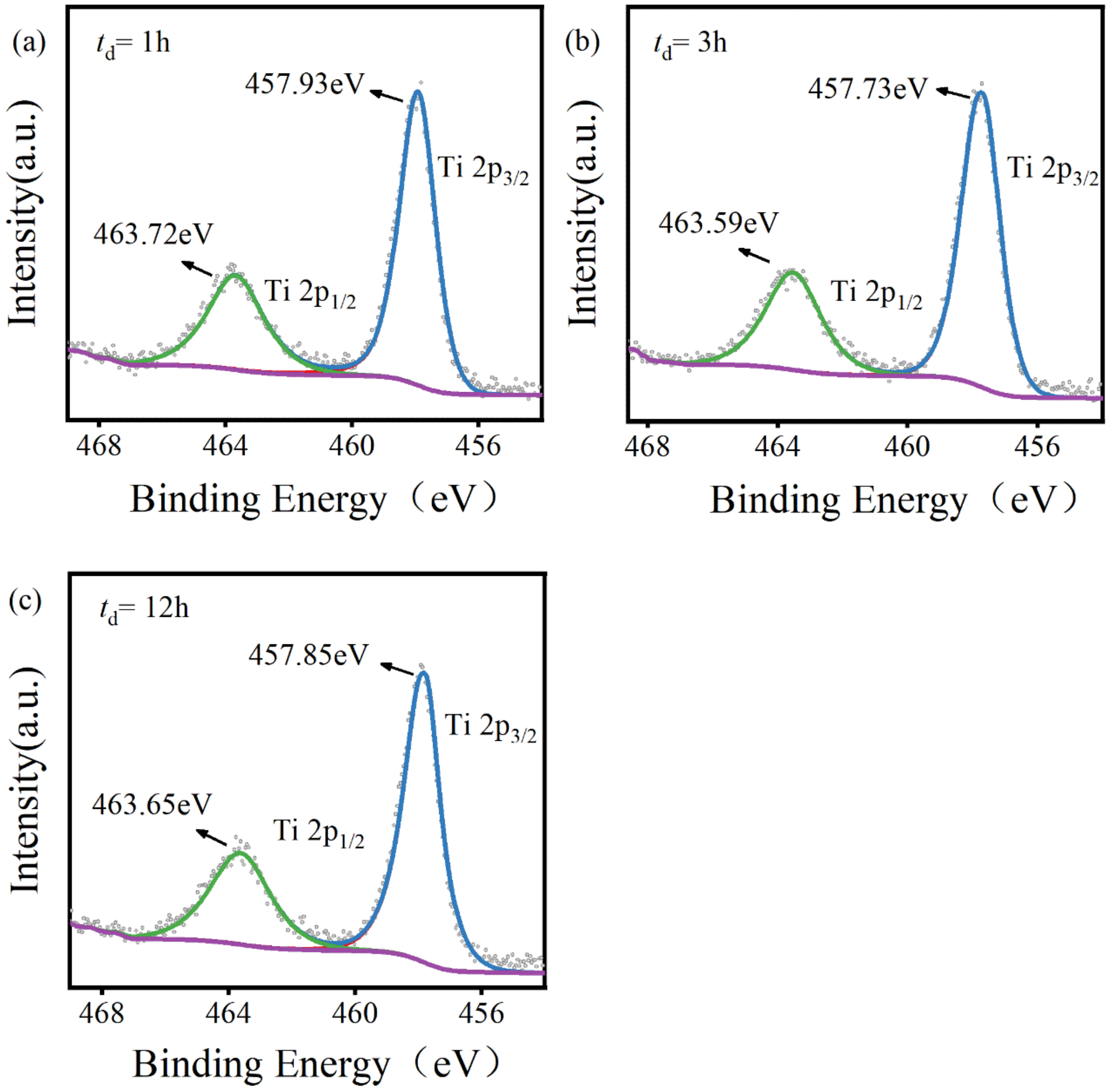

Figure S3. Ti 2p XPS spectra of BGTH7 ceramics sintered for (a) 1 h (b) 3 h (c) 12 h at 1400 °C

Well spin-orbital splitting peaks $Ti2p_{3/2}$ (at approximately 457.8 eV) and $Ti2p_{1/2}$ (at approximately 463.6 eV) were observed. And the expected spin-orbit splitting of 5.8 eV for a Ti^{4+} species was observed. These results confirm that Ti is in the Ti^{4+} $3d_0$ state in the BGTH7 ceramics sintered at 1400 °C.

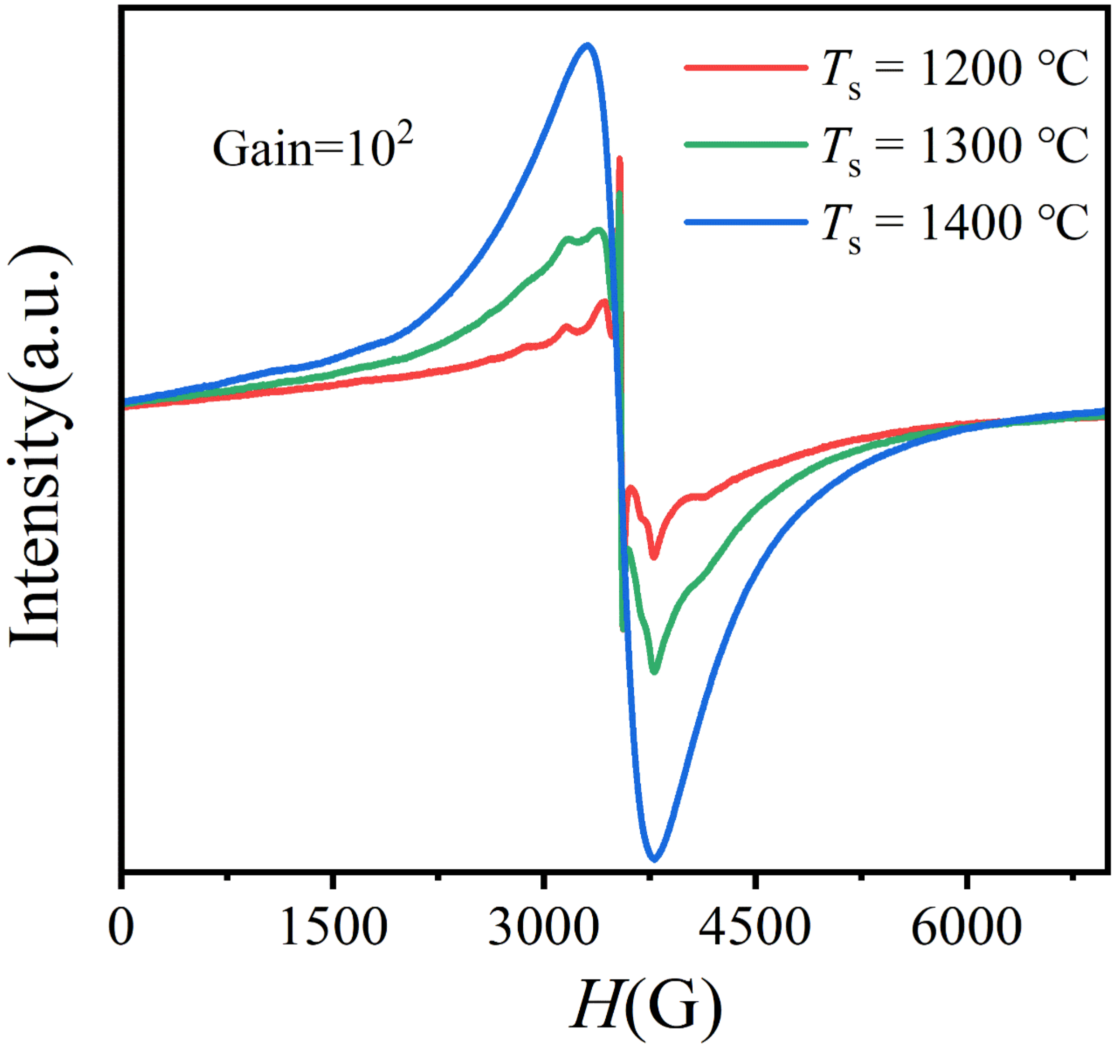

Figure S4. EPR spectra of BGTH7 ceramics sintered at different T_s for 12 h measured at room temperature.

The EPR spectrum of Gd^{3+} is highly dependent on the symmetry of ion coordination. The EPR spectrum of Gd^{3+} at room temperature shows a seven-tap signal when the ion coordination has low symmetry, as shown in the EPR spectrum of the BGTH7 ceramics sintered at T_s = 1200 °C and 1300 °C**.** For the high symmetry of ion coordination, the seven signals of Gd^{3+} present a broad signal, as shown in the EPR spectrum of the BGTH7 ceramics sintered at 1400 °C.

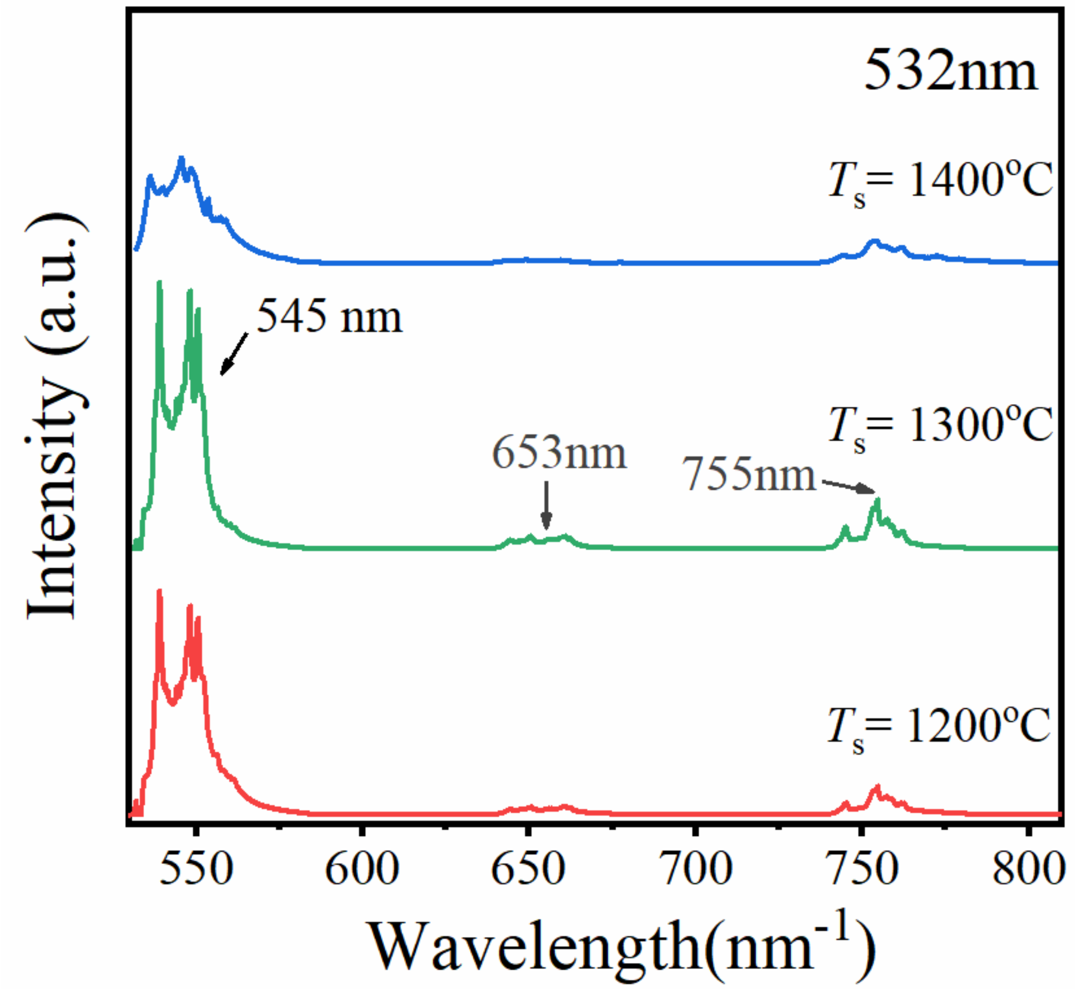

Figure S5. Raman spectra under 532 nm excitation of ceramics sintered at different T_s for 12 h measured at room temperature.

Due to the annihilation of the strong photoluminescence signal of Ho^{3+}, the Raman spectrum failed to show the optical characteristics of $BaTiO_3$ excited at 785 nm. Under 532 nm excitation, the transitions of $^5F_4/^5S_2 \rightarrow ^5I_8$ at 545 nm, $^5F_5 \rightarrow ^5I_8$ at 653 nm, and $^5F_4/^5S_2 \rightarrow ^5I_7$ at 755 nm are attributed to the occupation of the Ba site of Ho^{3+} ions.

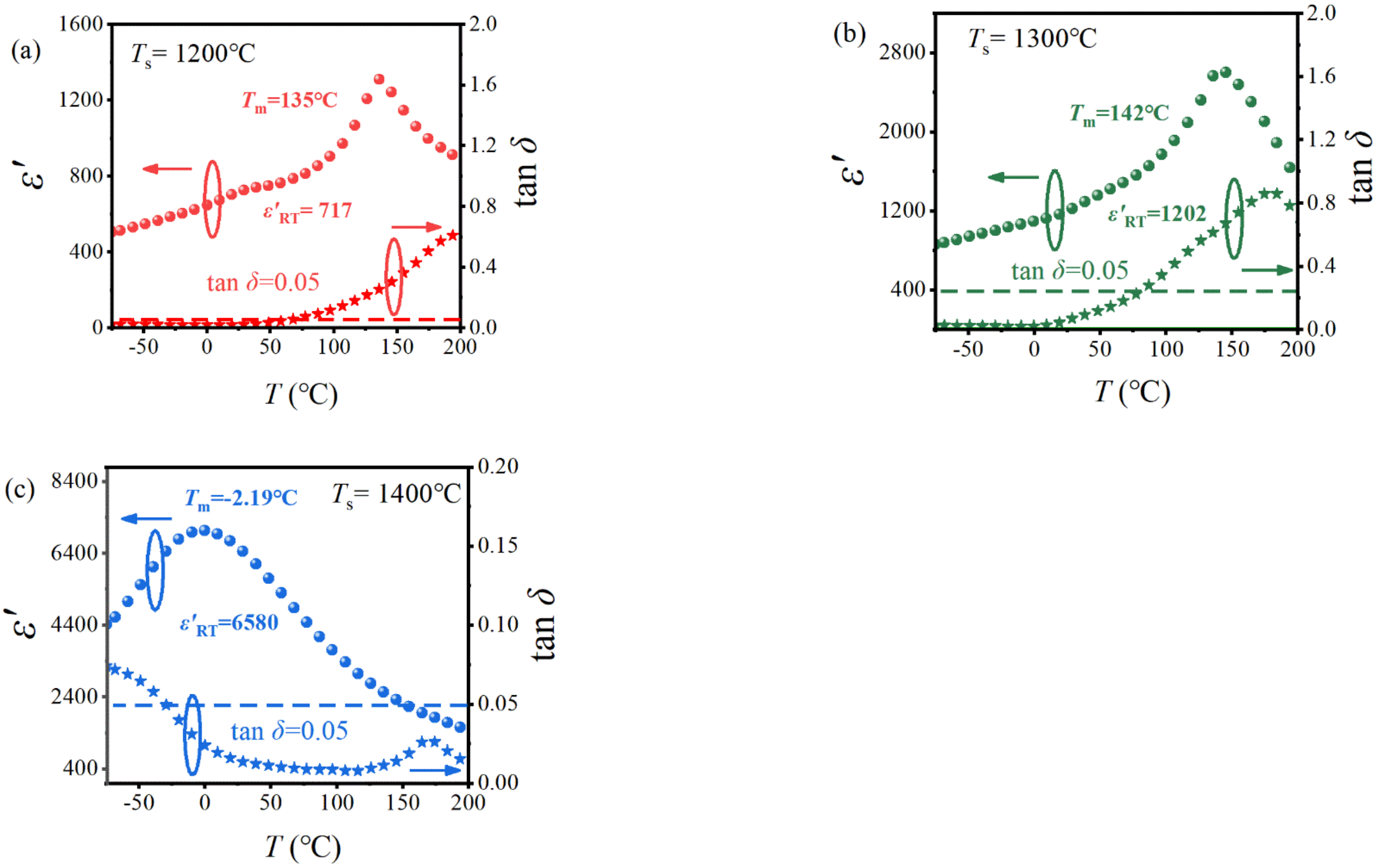

Figure S6. Temperature dependence of the dielectric constant (ε') and the dielectric loss (tan δ) for ceramics sintered at T_s = 1200 °C, 1300 °C and 1400 °C.

The BGTH7 ceramics sintered at T_s= 1200 °C and 1300 °C for 12 h exhibited low ε'_{RT} (ε'_{RT} < 1500), and high dielectric peak temperature (T_m) of 135 °C and 142 °C, respectively. The BGTH7 ceramics sintered at 1400 °C exhibited high ε'_{RT} (ε'_{RT} = 6580) with low dielectric loss (tan δ = 0.0144), which exhibited excellent dielectric performance.

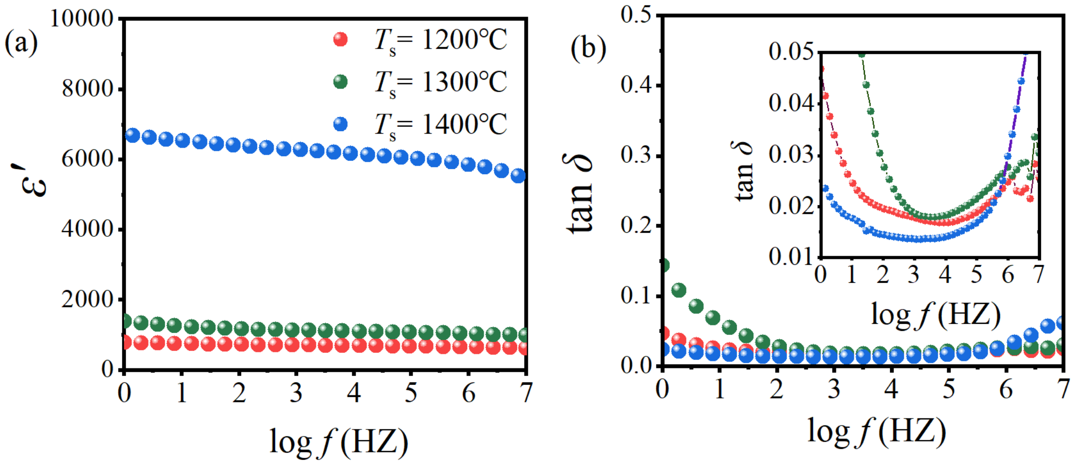

Figure S7. Frequency dependence of the dielectric permittivity (ε') and the dielectric loss (tan δ) for BGTH7 ceramics sintered at T_s = 1200 °C, 1300 °C and 1400 °C.

The ε' and tan δ of BGTH7 ceramics sintered at 1200 °C and 1300 °C remained stable with frequency. However, ε' was lower than BGTH7 ceramics sintered at 1400 °C.

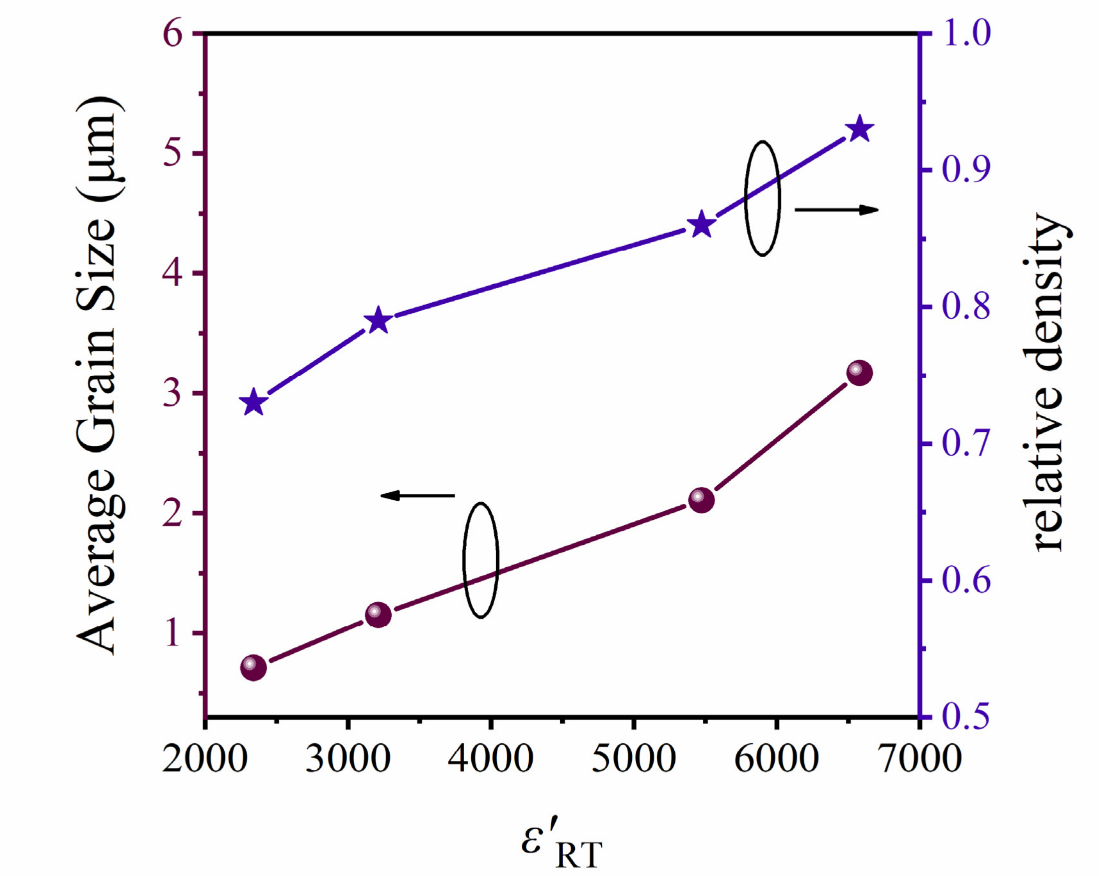

Figure S8. ε'_{RT} and tan δ at room temperature vary with grain size (GS) and relative density (ρ_r) for BGTH7 ceramics sintered at 1400 °C

ε'_{RT} gradually increases from 1762 to 6580 as the average grain size changes from 0.71 to 3.17 μm (ρ_r from 73% to 93%, respectively).

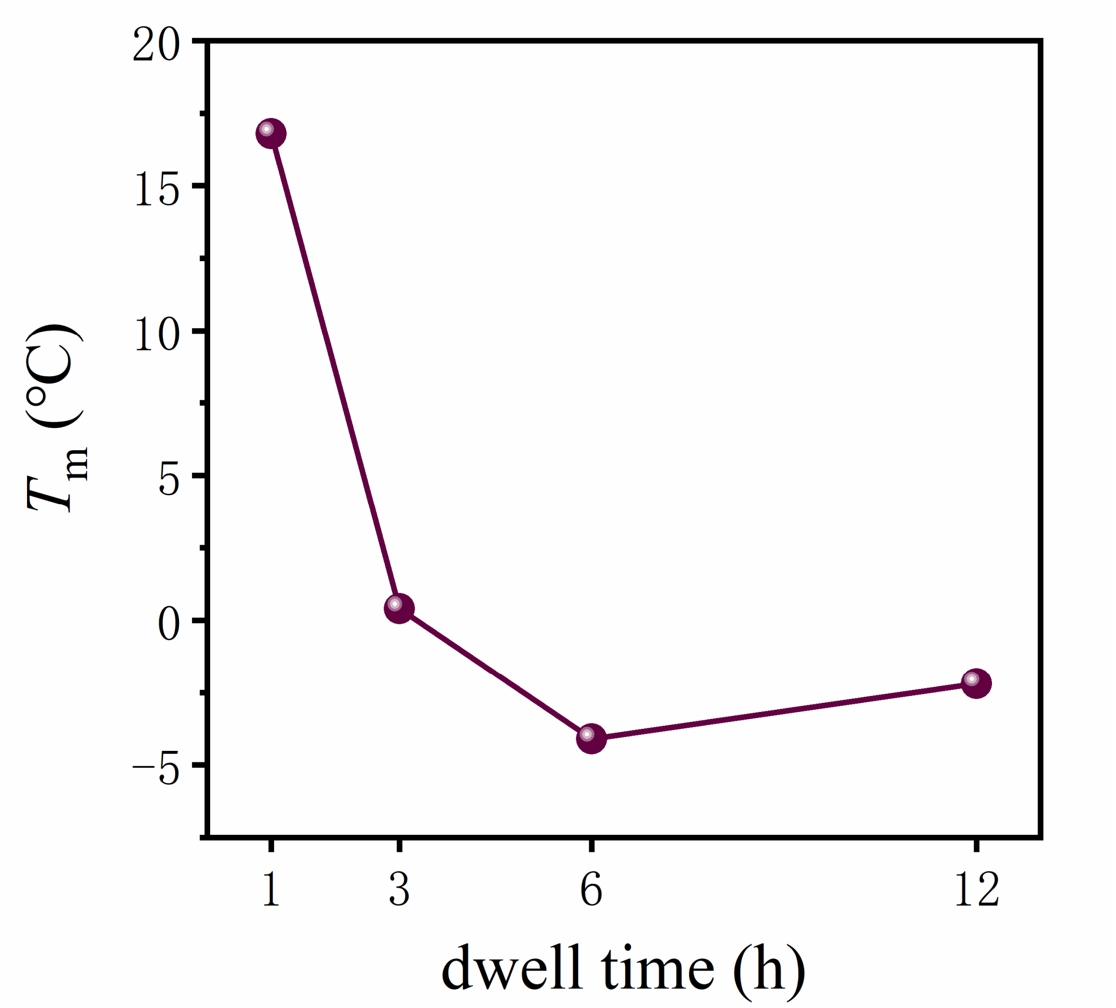

Figure S9. Dielectric peak temperature varies with dwell time for BGTH7 ceramics sintered at 1400 °C

T_m first decreased and then increased. When some Gd^{3+} ions are incorporated into Ba sites, the decrease in t shifts T_m to low temperature. Instead, the substitution of Ho^{3+} for Ti^{4+} and Ba^{2+} leads to T_m moving towards higher temperatures, similar to the incorporation of Ca^{2+}. In other words, the charge-coupled substitution of Gd^{3+} and Ho^{3+} at both Ba and Ti sites has different effects on T_m.

Table S1. Rietveld Refined lattice parameters of BGTH7 ceramics sintered at 1400 °C

Sintering conditions	*a, b* (Å)	*c* (Å)	*c*/*a*	V_0(Å^3)	*α, β, γ*
T_s =1400 °C, t_d = 1 h	4.0232(4)	4.0384(6)	1.00377(8)	65.36(8)	90°
T_s =1400 °C, t_d = 3 h	4.0238(3)	4.0314(4)	1.00189(3)	65.27(3)	90°
T_s =1400 °C, t_d = 6 h	4.0246(1)	4.0294(3)	1.00119(7)	65.26(6)	90°
T_s =1400 °C, t_d = 12 h	4.0247(2)	4.0293(3)	1.00114(5)	65.26(8)	90°

Table S2. Ionic radii as a function of coordinate number (CN)

Ions	CN	r (Å)
Ba^{2+}	12	1.61
Ti^{4+}	6	0.605
Gd^{3+}	12	1.215*
Gd^{3+}	6	0.938
Ho^{3+}	12	1.18
Ho^{3+}	6	0.901

The ionic radii denoted "*" were obtained by extrapolating method for Shannon radii.

The results of Rietveld refinement in Table S1 show precise lattice parameters ($a = b \neq c$, $\alpha = \beta = \gamma = 90°$) and a slight reduction in unit cell volume (V_0) with increasing t_d, confirming that all BGTH7 ceramics have a tetragonal phase structure. The radii of the Gd^{3+} and Ho^{3+} are between those of Ba^{2+} and Ti^{4+} as shown in Table S2.

Table S3. EIA Definition of Class II Capacitors.

The first letter		The second letter		The third letter	
Symbols	Lower temperature limit (°C)	Symbols	Upper limit temperature (°C)	Symbols	Maximum capacitance change rate
Z	+10	4	+65	A	±1.0
Y	-30	5	+85	B	±1.5
X	-55	6	+105	C	±2.2
		7	+125	D	±3.3
		8	+150	E	±4.7
		9	+200	F	±7.5
				P	±10.0
				R	±15.0
				S	±22.0
				T	+22/-33
				U	+22/-56
				V	+22/-82

The three letters indicate the lower operating temperature limit, the upper operating temperature limit, and the rate change of capacitance with temperature (based on the capacitance value at 25 °C). The rate change of capacitance can be calculated using the
following equation [1]:

$$\Delta C = (C - C_{RT}) / C_{RT} \quad (1)$$

where C is the capacitance at any operating temperature and C_{RT} is the capacitance at room temperature. For example, X8R indicates a capacitance change rate of $\Delta C \leq \pm$ 15% over the temperature range of − 55 to 150 °C. X7U indicates a capacitance change rate of − 22% ≤ $\Delta C \leq$ +56% over the temperature range of − 55 to 125 °C. X6U indicates a capacitance change rate of − 22% ≤ $\Delta C \leq$ +56% over the temperature range of − 55 to 105 °C [2, 3]. The capacitance can be calculated using the following equation [4]:

$$C = \varepsilon' S / 4\pi k d \quad (2)$$

The change in capacitance is equivalent to the change in dielectric constant when other conditions are certain. The rate of change of the dielectric constant can be obtained by using the following equation:

$$\Delta\varepsilon' = (\varepsilon' - \varepsilon'_{RT}) / \varepsilon'_{RT} \quad (3)$$

where ε' is the dielectric constant at any operating temperature and ε'_{RT} is the dielectric constant at room temperature.

Table S4. Dielectric properties of all BGTH7 ceramics

Sintering conditions	Specification	ε'_{RT}	tan δ_{RT}	T_m
T_s = 1200 ℃, t_d =12 h	X4S	717	0.0228	135 ℃
T_s = 1300 ℃, t_d =12 h	X4T	1202	0.0616	142 ℃
T_s = 1400 ℃, t_d =1 h	X8R	2340	0.0190	16.8 ℃
T_s = 1400 ℃, t_d =3 h	X7U	3213	0.0161	0.38 ℃
T_s = 1400 ℃, t_d =6 h	X7U	5475	0.0176	-4.12 ℃
T_s = 1400 ℃, t_d =12 h	X6U	6580	0.0144	-2.19 ℃

Table S5. The dielectric properties of this work are compared with those of previously reported $BaTiO_3$-based dielectric ceramics with X7U specifications.

Nominal formula	ε_{RT}'	ρ_r (%)	tan δ RT	Reference
$(Ba_{0.93}La_{0.07})(Ti_{0.93}Tb_{0.07})O_3$	2450	84%	0.03	[45]
$(Ba_{0.97}La_{0.03})(Ti_{0.97}Tb_{0.03})O_3$-0.09Tb	5990	96%	0.06	[45]
$Ba(Ti_{0.95}Y_{0.05})O_3$	5250	—	0.01	[46]
$(Ba_{0.95}Eu_{0.05})(Ti_{1-0.96}Cr_{0.04})O_3$	4550	94%	0.02	[47]
$0.1BiAlO_3$-$0.9BaTiO_3$	3300	93.8%	0.02	[48]
Ag-$BaTiO_3$-1100	5500	78%	0.02	[49]
N-$BaTiO_3$-10ZnO	2500	85%	0.02	[2]
$0.98BaTiO_3$-$0.02Sr_2CoMoO_6$	3300	—	0.01	[50]
$95BaTiO_3$-5(Bi_2O_3-BaO-P_2O_5)	1563	—	0.01	[51]
$0.94BaTiO_3$-$0.06BiFeO_3$	1700	—	0.01	[52]
BT@0.8(0.25BZT-0.75BT）	3000	—	0.05	[53]
$0.96BaTiO_3$-$0.04Bi(Li_{1/3}Zr_{2/3})O_3$	2000	—	0.04	[54]
Cold Sintering-AN-S60	2178	93%	0.01	[55]

0.96(0.94BNT-0.06BT)-0.04SBN	2000	—	0.05	[56]
This work	5474	93%	0.02	

References

[1] MJ, Pan, CA, Randall, Ieee. Electr. insul. M. **26**, 44 (2010).
[2] A.W. Tavernor, H-P S. Lia, R. Stevens, J. Eur. Ceram. Soc. **19**, 1859 (1999).
[3] O.Furukawa, M. Harata, M. Imai, Y. Yamashita, S. Mukaeda Low firing and high dielectric constant X7R ceramic dielectric for multilayer capacitors based on relaxor and barium titanate composite, J. Mater. Sci. **26**, 5838 (1991).
[4] T. Hino, N. Matsumoto, M. Nishida, T. Araki, Appl. Surf. Sci. **254**, 2638 (2008).
